# Predicting Risk of Infection in Patients with Newly Diagnosed Multiple Myeloma: Utility of Immune Profiling

**DOI:** 10.3389/fimmu.2017.01247

**Published:** 2017-10-05

**Authors:** Benjamin W. Teh, Simon J. Harrison, Cody Charles Allison, Monica A. Slavin, Tim Spelman, Leon J. Worth, Karin A. Thursky, David Ritchie, Marc Pellegrini

**Affiliations:** ^1^Department of Infectious Diseases, Peter MacCallum Cancer Centre, Parkville, VIC, Australia; ^2^Sir Peter MacCallum Department of Oncology, Peter MacCallum Cancer Centre, Melbourne, VIC, Australia; ^3^Walter and Eliza Hall Institute, Parkville, VIC, Australia; ^4^National Centre for Infections in Cancer, Parkville, VIC, Australia; ^5^Department of Haematology, Victorian Comprehensive Cancer Centre, Parkville, VIC, Australia; ^6^Department of Medicine, University of Melbourne, Parkville, VIC, Australia; ^7^Victorian Infectious Diseases Service, Doherty Institute for Infection and Immunity, Parkville, VIC, Australia; ^8^Department of Medical Biology, University of Melbourne, Parkville, VIC, Australia

**Keywords:** prediction, infection, risk, immune, profiling, myeloma

## Abstract

**Background:**

A translational study in patients with myeloma to determine the utility of immune profiling to predict infection risk in patients with hematological malignancy was conducted.

**Methods:**

Baseline, end of induction, and maintenance peripheral blood mononuclear cells from 40 patients were evaluated. Immune cell populations and cytokines released from 1 × 10^6^ cells/ml cultured in the presence of a panel of stimuli (cytomegalovirus, influenza, *S. pneumoniae*, phorbol myristate acetate/ionomycin) and in media alone were quantified. Patient characteristics and infective episodes were captured from clinical records. Immunological variables associated with increased risk for infection in the 3-month period following sample collection were identified using univariate analysis (*p* < 0.05) and refined with multivariable analysis to define a predictive immune profile.

**Results:**

525 stimulant samples with 19,950 stimulant–cytokine combinations across three periods were studied, including 61 episodes of infection. Mitogen-stimulated release of IL3 and IL5 were significantly associated with increased risk for subsequent infection during maintenance therapy. A lower Th1/Th2 ratio and higher cytokine response ratios for IL5 and IL13 during maintenance therapy were also significantly associated with increased risk for infection. On multivariable analysis, only IL5 in response to mitogen stimulation was predictive of infection. The lack of cytokine response and numerical value of immune cells were not predictive of infection.

**Conclusion:**

Profiling cytokine release in response to mitogen stimulation can assist with predicting subsequent onset of infection in patients with hematological malignancy during maintenance therapy.

## Introduction

With recent advances in immune-based therapies, hematological malignancies such as plasma cell malignancy multiple myeloma (MM) have been transformed into chronic diseases maintained by multiple lines of therapy (1, 2). Therapy with immunomodulatory drugs (IMiDs), proteasome inhibitors (PI), and autologous stem cell transplantation (ASCT) for eligible patients are the current standard of care for MM (2). These therapies have effects on the immune system ranging widely from immune activation to immune suppression, a paradigm shift from the predictable myelosuppression seen with conventional chemotherapy. With inevitable disease progression, treatment phases are repeated to obtain and maintain disease response resulting in unmeasurable cumulative immunosuppression (3).

Infections remain a leading cause of morbidity and mortality in these patients with nearly 50% of early deaths due to infection (4). Identified clinical risk factors for infection in MM patients range from receipt of intensive combination systemic chemotherapy, cumulative doses of corticosteroids to number of lines of therapy (5). In addition, there is differential infection risk posed by IMiDs and PI with varying treatment periods (3). Therefore, clinical risk assessment for infections has become increasingly complex and unreliable due to unpredictable interaction between patients, disease, and treatment-related risk factors. With further advances in immunotherapy for a range of hematological malignancies including MM, infective risk prediction will become even more challenging (6–9).

Comprehensive functional and numerical measurement of the immune system or “immune profiling” could assist with predicting risk of infection, a common management issue across hematology patient groups ranging from those receiving monoclonal antibody therapy to those receiving conventional chemotherapy. Its role and use in the management of infection in hematology patients remains undefined. Therefore, we conducted an exploratory translational study to assess the utility of immune profiling as a means of signaling future risk for infection in a population of MM patients undergoing a standardized treatment regimen as part of a clinical trial.

## Materials and Methods

### Patient Population and Definitions

Patients with newly diagnosed MM enrolled in a clinical treatment trial at Peter MacCallum Cancer Centre (PMCC) were evaluated (Australia New Zealand clinical trials registry number 12613000344796). In brief, patients received induction with lenalidomide and dexamethasone (0–40 mg weekly), ASCT 4–6 weeks following completion of induction chemotherapy and maintenance with lenalidomide (25 mg, days 1–21) commencing 4–5 weeks posttransplant with a cohort of 10 patients receiving autologous dendritic cell vaccination with primary MM cell lysate (up to six courses). Blood samples were collected prospectively at multiple defined time points from patients who participated in this trial (Figure S1 in Supplementary Material).

The following prophylactic measures were routinely used at our institution: (i) valaciclovir or aciclovir prophylaxis for patients undergoing ASCT (up to 3 months post-ASCT), (ii) fluconazole prophylaxis for the period of ASCT (up to neutrophil recovery), and (iii) trimethoprim–sulfamethoxazole prophylaxis for patients receiving more than 20 mg prednisolone equivalent for more than 4 weeks or in the setting of known intensive immunosuppression, such as following ASCT. Of note, fluoroquinolone prophylaxis was not used during periods of neutropenia.

For the purpose of this study, timing of samples was defined as follows: “Baseline” refers to period prior to commencement of MM therapy up to cycle 2 of induction, “End of induction (EOI)” refers to period following completion of induction therapy and prior to stem cell collection. “Maintenance” refers to period coinciding with cycle 2 of maintenance therapy. These time points reflect clinical assessment and treatment periods and were chosen to ensure similar time periods between samples.

Clinical and microbiology records were reviewed to capture patient demographics, MM characteristics, and characteristics of infective episodes. Episodes of infection were defined and classified as microbiologically (MDI), clinically defined infections (CDI), or fever of unknown focus (FUF) and graded according to internationally published criteria (10–13). MM disease response was defined according to international myeloma working group criteria (14).

As a measure of the ability of immune cells to mount a cytokine response, a cytokine response ratio as defined by the ratio of cytokine values for PMA-stimulated samples to values for unstimulated samples was determined. T-helper 1/T-helper 2 (Th1/Th2) ratio was defined by the ratio of cytokine values of IFNγ to IL4. The Peter MacCallum Cancer Centre and Royal Melbourne Hospital’s human research ethics committees approved this research. All subjects in the clinical trial gave written informed consent in accordance with the Declaration of Helsinki.

### Sample Collection and Analysis

Patient samples were obtained at baseline, EOI, and during maintenance phase. Total neutrophil count in patient samples was established at the time of sample collection using an automated cell counter (Cell Dyn Sapphire, Abbott Diagnostics). Peripheral blood mononuclear cells (PBMCs) were isolated by Ficoll density separation and stored in RPMI/FBS/10%DMSO (Fetal bovine serum, dimethyl sulfoxide) at −40°C prior to analysis. PBMCs were rested in RPMI supplemented with 10% (vol/vol) FBS for 24 h after rapid thawing, and immune cell population data was collected using the fluorescence-activated cell sorting LSR Fortessa X20 (BD Biosciences, USA), and analyzed using FlowJo (Treestar). The following labeled monoclonal antibodies were used to measure the number of circulating CD4+ and CD8+ T cells, B cells, DCs, NK cells, and monocytes; anti-CD3, CD4, CD8, CD14, CD16, CD19, CD45, CD56, CD57, HLA-DR, CD11c (all BD Biosciences).

2 × 10^5^ cells were cultured in 200 µl medium on U-bottom 96-well plates in the presence of either four stimuli; cytomegalovirus (CMV-Merlin), influenza (X31), *S. pneumoniae* (Serotype 19f), phorbol myristate acetate/ionomycin (PMA), and also in media alone (unstimulated) for 72 h. The stimuli used were whole UV-inactivated CMV, UV-inactivated influenza, heat-killed *S. pneumoniae* (SP) (all at equivalent multiplicity of infection of 1), and PMA/Iono (50 ng/ml PMA, 250 nM ionomycin; Sigma, St. Louis, MO, USA). After 72 h, culture supernatants were harvested and assayed using the Milliplex human cytokine bead array (EMD Millipore Corporation, MA, USA) run on the Bio-plex 200 system (Bio-Rad Laboratories, USA) for the following cytokines: EGF, FGF, Eotaxin, TGF-α, G-CSF, Flt-3L, GM-CSF, Fractalkine, IFN-α2, IFN-γ, GRO, IL-10, MCP-3, IL-12P70, MDC, IL-12P40, IL-13, IL-15, scd40L, IL-17A, IL-1RA, IL-1α, IL-9, IL-1β, IL-2, IL-3, IL-4, IL-5, IL-6, IL-7, IL-8, IP-10, MCP-1, MIP-1α, MIP-1β, TNFα, TNFβ, VEGF, PDGF-AA, PDGF-AB/BB, and RANTES.

### Data Analyses

Cytokine concentrations were calculated using Bio-Plex Manager 5.0 software (Bio-Rad Laboratories, USA) with a five parameter curve-fitting algorithm applied for standard curve calculations. Minimum detectable concentration was calculated using MILLIPLEX Analyst 5.1 (Millipore, EMD corporation) with the range of minimum detectable concentration for each cytokine summarized in Figure S2 in Supplementary Material. The same standards, positive and negative controls were used across all batches and the manufacturer’s inter-assay variability is summarized in Figure S3 in Supplementary Material. For cytokine values detected above or below the calculated range, a value of 1 log above or below the highest or lowest reported value was assigned for statistical purposes, as these samples remained biologically relevant.

### Statistical Analyses

Categorical variables were summarized using frequency and percentage. Incidence of infection in 3-month period following sample collection was chosen as the outcome of interest to evaluate the ability of immune profiles to signal increased risk for infection. For statistical analysis, only the first observed infection event per patient for the defined period was included in the analysis. Immunological variables (immune cell numbers, cytokine–stimulant combinations, cytokine response ratio) were evaluated using univariate analysis to quantify their association with incidence of infection and difference between patients with and without infection in a 3-month period (Rank-sum testing for non-parametric data). A *p* < 0.05 was considered significant and used to define an immune profile. Bonferroni correction was then performed to account for multiple comparisons. Multivariable Firth’s logistic regression was performed to adjust observed associations for clinical variables (confounders). The Youden and Liu method for cut-point analysis of receiver operator curves (ROC) was used to define the value that best predicts infection for immune variables identified. All analyses were performed using Stata (version 13.1, StataCorp Inc., College Station, TX, USA).

## Results

### Characteristics of Patients and Disease Response

Forty patients were eligible for the current study. Patients had a median age of 53.1 years [Interquartile range (IQR) 48.8–61.3 years], 62.5% were males and the median Charlson comorbidity score was 5.0 (IQR 4–5). The majority of patients had IgG MM (55.5%) with 60.0% international staging system stage 1 disease. Patients were evaluated for disease response at the EOI and commencement of maintenance therapy and is summarized in Table 1.

**Table 1:** Summary of disease response by sample period.

Disease response	End of induction	Maintenance
	
	*N* = 38	*N* = 28
Complete response	0 (0.0%)	7 (25.0%)
Partial response	22 (57.9%)	19 (67.9%)
Minimal response	2 (5.3%)	0 (0.0%)
Stable disease	3 (7.9%)	0 (0.0%)
Progressive disease	0 (0.0%)	1 (3.6%)
Not assessed	11 (28.9%)	1 (3.6%)

### Characteristics of Samples and Infection

Overall, there were 525 stimulant-samples with 19,950 stimulant–cytokine combinations. There were 195 stimulant-samples from 39 patients at baseline, 190 at EOI (38 patients), and 140 (28 patients) during maintenance therapy. Insufficient samples were available for 1, 2, and 12 patients at baseline, EOI, and maintenance respectively. Of 39 patients, 16 patients (41.0%) developed an infection in the 3 months following baseline sample collection. 36 patients (94.7%) and 9 patients (32.1%) developed an infection in the 3 months following EOI and maintenance periods respectively. The category and severity of infection by sample period is summarized in Table 2. There was no significant difference in the rates of infection between patients who did (30.0%) or did not receive DC vaccination during maintenance therapy (33.3%, *p* = 0.87).

**Table 2:** Category and severity of infection by sample period.

	Baseline	End of induction	Maintenance
	
Episodes of infection	16	36	9
**Categories of infection**			
Microbiological-diagnosed	2 (12.5%)	14 (38.9%)	0 (0.0%)
Bacterial	1	10	
Viral	1	2	
Fungal	0	2	
Clinically diagnosed	9 (56.3%)	4 (27.8%)	8 (88.9%)
Respiratory	7	1	6
Skin and soft tissue	1	3	0
Urinary tract	1	0	0
Gastrointestinal	0	0	2
Fever of unknown focus	5 (31.3%)	18 (50.0%)	1 (11.1%)
**Severity of infection**			
Grade 1 and 2	9 (56.3%)	1 (2.8%)	8 (88.9%)
Grade 3	2 (12.5%)	1 (2.8%)	0 (0.0%)
Grade 4≥	5 (31.3%)	34 (94.4%)	1 (11.1%)

### Identification of Immune Profile That Best Predicts Infection

The following stimulant–cytokine combinations were associated with risk of infection in the following 3-month period with *p* < 0.05 on univariate analysis: unstimulated-IL5 at EOI was associated with reduced risk of infection while increased risk was seen with PMA-IL3 and PMA-IL5 in maintenance samples. However, the result for EOI is likely to be unreliable due to the small number of non-infection events in this cohort (*n* = 2). The median cytokine values by infection status and details of risk per unit increase in cytokine value (picogram per milliliters) on univariate analysis are summarized in Table 3. Correcting for multiple comparisons, only PMA-IL5 remained significantly associated with risk for infection (Table S1 in Supplementary Material).

**Table 3:** Immune parameters associated with risk of infection, by stimulant–cytokine combination and sample period.

Sample period	Stimulant	Cytokine	Episodes of infection *N* (%)		Cytokine values (median, pg/ml)		Odds ratio^a^	*P-*value
			Infection	No infection	Infection	No infection		
EOI	Unstimulated	IL5	36 (94.7)	2 (5.3)	0.29	0.59	0.0002	0.04
Maintenance	PMA	IL3	9 (32.1)	19 (67.9)	890.70	193.40	1.001	0.04
	PMA	IL5	9 (32.1)	19 (67.0)	404.40	62.70	1.010	0.01

*^a^Per unit increase in cytokine in picogram per milliliter*.

*EOI, end of induction; PMA, phorbol myristate acetate/ionomycin*.

As not all patients with FUF will have an infection, an analysis was performed with only MDI and CDI as the outcome of interest. In this analysis, only PMA-IL5 during maintenance was significantly associated with infection after adjustment for multiple comparisons. The median value of PMA-IL5 was 405.43 for patients with subsequent infection compared to 66.69 pg/ml (*p* = 0.0003). This analysis is summarized in Table S2 in Supplementary Material.

Neutrophil count, B cells, CD4, CD8, NK cells, DC cells, and monocytes were evaluated and no immune cell parameter was associated with increased risk for infection. Median cell numbers by infection status and sample period are summarized in Table 4.

**Table 4:** Summary of immune cell counts by infection status and sample period.

Immune cells	Infection (×10^6^/ml) median	No infection (×10^6^/ml) median	*P-*value
**Baseline**			
Neutrophils	3.10	2.14	0.05
B cells	0.09	0.09	0.81
CD4+ cells	0.53	0.75	0.49
CD8+ cells	0.18	0.26	0.43
NK cells	0.05	0.06	0.73
DC	0.01	0.02	0.90
Monocytes	0.04	0.03	0.83
**End of induction**			
Neutrophils	2.73	1.45	0.28
B cells	0.03	0.04	0.95
CD4+	0.69	0.54	0.70
CD8+	0.22	0.05	0.18
NK cells	0.10	0.07	0.98
DC	0.01	NA	NA
Monocytes	0.06	NA	NA
**Maintenance**			
Neutrophils	1.61	1.63	0.37
B cells	0.09	0.07	0.68
CD4+ cells	0.28	0.40	0.42
CD8+ cells	0.29	0.38	0.53
NK cells	0.05	0.06	0.87
DC	0.01	0.00	0.13
Monocytes	0.16	0.12	0.43

*NA, not available*.

Immune profiles that best predict infection during a 3-month period on univariate analysis was identified to be mitogen-stimulated PMA-IL3 and PMA-IL5 for maintenance samples.

### Determination of Cytokine Values that Best Predict Infection

**Figure 1:**
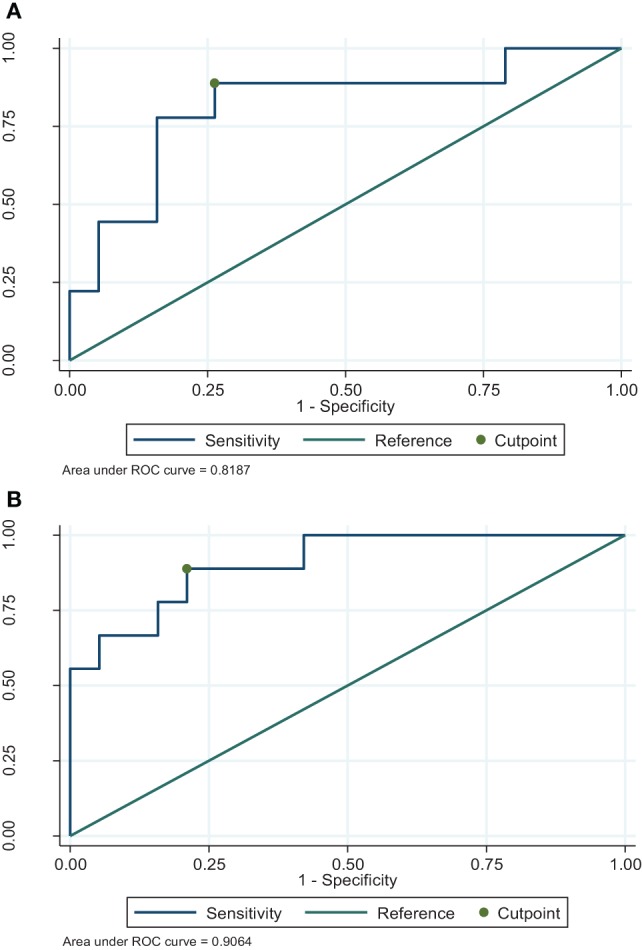
Receiver operating characteristic curves for optimal cut-point to determine risk for infection for stimulant–cytokine combinations during maintenance. **(A)** Maintenance phorbol myristate acetate/ionomycin (PMA)-IL3. **(B)** Maintenance PMA-IL5.

For the identified immune profiles, optimal values that best predict infection were determined using the Youden and Liu method of ROC analysis. For unstimulated-IL5 samples at EOI, optimal cut-off was not reliable due to low number of patients without infection (5.3%). For maintenance period, optimal cut-off value for IL3 and IL5 in PMA (mitogen) stimulated samples were 351.0 and 178.0 pg/ml, respectively. Both had a sensitivity of 89.0% for detection of 3-month risk for infection at the specified cut-offs. The ROC curves for these cytokines are presented in Figure 1. Table 5 summarizes the optimal predictive cytokine values, their sensitivity, and specificity and accuracy for identified immune profiles.

**Table 5:** Optimal predictive value for infection for identified immune profiles by sample period.

Period	Immune profile		Optimal predictive value (pg/ml)	Sensitivity (%)	Specificity (%)	Diagnostic accuracy
	Stimulant	Cytokine				
Maintenance	PMA	IL 3	351.0	89.0	74.0	0.81
	PMA	IL 5	178.0	89.0	79.0	0.84

*PMA, phorbol myristate acetate/ionomycin*.

### Determination of Cytokine Responses That Best Predict Infection

Cytokine response ratios were evaluated for patients with and without subsequent infection and only cytokine response ratios for IL5 and IL13 were significantly associated with increased risk for infection (*p* = 0.01 for both). Correcting for multiple comparisons, these ratios do not remain significant. A summary of these response ratios is provided in Table 6.

**Table 6:** Summary of significant cytokine response ratio (mitogen-stimulated:unstimulated samples) in patients with and without infection, by sample period.

Sample period	Stimulant	Cytokine	Cytokine response ratio^a^		Odds ratio	*P-*value
			Infection	Non-infection		
Maintenance	PMA:unstimulated	IL 5	25,063	3,793	1.0001	0.01
	PMA:unstimulated	IL 13	163,903	10,509	1.0002	0.01

*PMA, phorbol myristate acetate/ionomycin*.

*^a^Ratio of cytokine values (pg/ml) of PMA-stimulated samples to cytokine values (pg/ml) from unstimulated (control) samples*.

Only PMA-stimulated maintenance samples had Th1/Th2 ratios that were significantly associated with infection. Th1/Th2 ratio was lower at 20.13 for patients with subsequent infection compared to a ratio of 92.00 for patients who did not develop infection (*p* = 0.03). The odds ratio was 0.97 (95% CI 0.94–0.99, *p* = 0.04) per unit increase in Th1/Th2.

### Refining the Predictive Immune Profile

There was no significant association found between immune markers evaluated and assessed disease response on univariate analysis across all sample periods (all *p* ≥ 0.05). As the study was conducted in a homogeneous population of newly diagnosed patients receiving standardized treatment, multivariable Firth’s logistic regression was used to account for myeloma disease response in maintenance samples and only PMA-IL5 remained significantly associated with increased risk of subsequent infection (*p* < 0.01). Accounting for receipt of DC vaccination during maintenance, only PMA-IL5 remained significantly associated with increased risk (*p* < 0.01). Cytokine response ratios and Th1/Th2 ratios were not significantly associated with increased risk after accounting for disease response and DC vaccination.

## Discussion

With increasing unreliability of clinical risk prediction due to complex interactions between treatment, patient, and disease-related factors, new approaches to determining risk of infection in patients with MM are required. Improved non-subjective infection risk assessment allows better targeting of prevention and prophylaxis measures. This is the first ever study profiling the numerical and functional responses of the adaptive and innate immune system as a means of predicting future risk of infection in patients with a hematological malignancy.

In our translational study, we found release of several key cytokines such as IL3 and 5 in response to PMA to be the most predictive of subsequent 3-month risk of infection with defined optimal cytokine values. Responses to pathogenic antigens such as CMV, influenza, and *S. pneumoniae* were not associated with increased risk. This suggests measurement of response to a pan-antigen (mitogen) such as PMA is sufficient to predict future risk, simplifying translation of its use to clinical practice. In contrast, other groups have reported cytokine releases to other antigens such as anti-CD3/CD28 and CMV/EBV lysates to be predictive, but this required the subsequent development of complex cytokine scores to be clinically useful (15).

This is the first study to report an association between IL3, malignancy, and risk of infection. In maintenance samples, higher values of IL 3 in response to PMA were associated with higher risk for infection in a 3-month period. IL 3 is a cytokine known for regulating the differentiation and expansion of hematopoeitic stem cells and promoting leukocyte survival and recently, animal studies suggest that IL3 may play a role in triggering the cytokine storm that mediates septic shock by inducing emergency hematopoiesis (16). Higher levels of IL3 (above 89.4 pg/ml) were associated with poorer prognosis from sepsis (16). We found a value of 351.0 pg/ml predicted risk of infection with a sensitivity of 89.0%, specificity 74.0%, and accuracy of 0.81. Higher test sensitivity enables identification of the largest possible population of patients at high risk of infection to target interventions such as prophylaxis or intensive surveillance. However, it should be noted that the performance (sensitivity and specificity) of these tests might vary in a validation cohort of patients.

This study has found an association between Th2 cytokines and increased risk for subsequent infection. Higher levels of IL 5 in response to mitogen stimulation were significantly associated with greater risk of infection in the 3-month period following maintenance sample collection. This remained significant even after accounting for disease response to treatment, a key clinical variable, on multivariable regression analysis. In addition, we found a higher relative production of Th2 cytokines (IL5, IL13) in response to mitogen stimulation (expressed as cytokine response ratio) rather than the lack of cytokine response to be associated with increased risk for infection. The dominance of a Th2 response in patients who are at higher risk of infection was confirmed by the significantly lower Th1/Th2 ratio as represented by IFNγ/IL4 levels seen on univariate analysis.

The association of Th2 cytokines IL4 and IL5 with MM is well established (17–19). In patients with untreated and relapsed MM, higher levels of Th2 cytokines compared to controls have been reported with close association with disease status and higher disease stage (17–19). The dominance of this anti-inflammatory cytokine environment, which suppresses antitumor immune activity, also results in impairment of pro-inflammatory cytokine-induced (e.g., IL 12, TNFα) cytotoxic T cell responses to pathogens (18, 20). It predisposes patients to infection and suggests a potential association between underlying disease activity and increased risk for infection. As previously reported, we comprehensively evaluated clinical factors associated with increased risk for infection and no clinical factor associated with increased risk during maintenance therapy was found (5). Therefore, measurement of Th2 cytokines in response to mitogen stimulation, in particular, IL5 could potentially be a useful predictive marker of future risk for infection in patients with MM especially during maintenance therapy.

In our study of patients managed with IMiD-based therapy and ASCT, immune cell numbers (neutrophils, CD4, CD8, NK, DC, B cells, and monocytes) were not associated with increased risk of subsequent infection across treatment periods. In contrast, others have reported lower CD4+ cell counts in patients with myeloma who develop opportunistic infection following intensive combination conventional therapy (21). While low numbers of myeloid cells such as neutrophils are classically associated with short-term risk of infection with invasive bacterial and fungal infections, enumeration of immune cells appears to be less useful in predicting long-term (3-month) risk of infection in patients managed with current generation IMiDs (2, 22, 23). A similar observation was also noted in clinical trials of next generation IMiDs. The majority of infection episodes reported were not associated with neutropenia despite nearly 50% of patients experiencing treatment-related neutropenia (24).

Limitations of our study include the focus upon newly diagnosed ASCT-eligible MM patients and the homogenous nature of treatment delivered. This highly selected trial patient population may limit immediate generalizability of findings to other patient populations. However, the benefit of studying this population was that consistency of anti-MM management minimized confounding effects of differential therapy and patient characteristics on observed immune patterns, removing the need to account for multiple clinical variables and allowing us to refine the panel of 19,950 cytokine–stimulant combinations to one key profile. Infectious outcomes were restricted to a 3-month period following sample collection, thus limiting evaluation of infective risk prediction beyond this period.

In our exploratory analysis of immune variables associated with increased risk of infection in patients with MM, a Th2-dominant cytokine response to mitogen appears to be associated with increased risk. We identified IL-5 in response to PMA antigen stimulation as a key immune profile and defined optimal values for this profile, to assist with predicting risk of subsequent onset of infection. Validation of this profile in prospective patient cohorts will further define its clinical utility and applicability to a wider range of hematological malignancies.

## Ethics Statement

This research was carried out in accordance with the recommendations of Peter MacCallum Cancer Centre and Royal Melbourne Hospital Human research ethics committees (HREC). All subjects in the clinical trial gave written informed consent in accordance with the Declaration of Helsinki. The clinical trial protocol was approved by the Peter MacCallum Cancer Centre HREC.

## Author Contributions

All authors were involved in the conception and design of the study. BT performed the research, data analysis, and together with TS performed the statistical analysis. CA assisted with performance of laboratory analysis. BT drafted the manuscript with input from all authors. All authors critically reviewed the manuscript and approved the final version for submission.

## Conflict of Interest Statement

SH has received research funding and honoraria from Celgene, Novartis, Amgen, Takeda, Sanofi and Janssen Cilag and research funding from Abbvie. MS have received research funding and honoraria from Pfizer, Gilead, and Merck Sharpe and Dohme. The Walter and Eliza Hall Institute of Medical Research had a research license agreement with TetraLogic Pharmaceuticals. MP provided consultative advice to, and was on the scientific advisory board of, TetraLogic Pharmaceuticals. All other authors declare that the research was conducted in the absence of any commercial or financial relationships that could be construed as a potential conflict of interest.
