# Supplementary material for: Predicting Risk of Infection in Patients with Newly Diagnosed Multiple Myeloma: Utility of Immune Profiling
Source: Front Immunol. 2017 Oct 5;8:1247. doi: 10.3389/fimmu.2017.01247 (PMC5633726; doi:10.3389/fimmu.2017.01247)
Supplement: Supplementary file 1 [file data_sheet_1.docx]

**Supplementary Figure 1: Standardised treatment regimen and timing of sample collections**


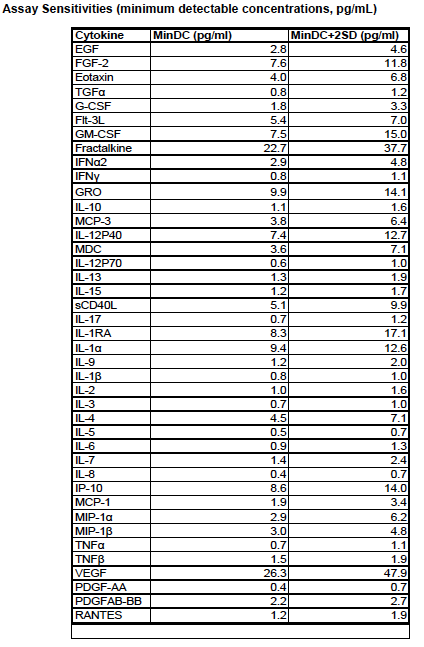


**Supplementary figure 2: Manufacturer reported minimum detectable concentration of each measured cytokine (pg/ml)**


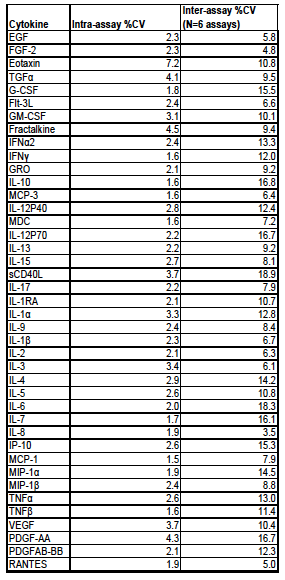


**Supplementary figure 3: Manufacturer reported inter-assay variability for each measured cytokine**

| **Sample period** | **Stimulant** | **Cytokine** | **Cytokine values (pg/ml)** | | ***P* value** |
| --- | --- | --- | --- | --- | --- |
|  |  |  | **Infection** | **Non-infection** |  |
| Baseline | Unstimulated | IL12P40 | 0.01 | 4.74 | 0.03 |
|  | Unstimulated | VEGF | 7.18 | 18.29 | 0.03 |
|  | PMA | IL2 | 16573.09 | 875380.00 | 0.02 |
|  | SP | MIP1α | 30.10 | 67.73 | 0.02 |
| EOI | Unstimulated | IL5 | 0.29 | 0.59 | 0.03 |
| Maintenance | Unstimulated | MCP1 | 9152.60 | 7454.80 | 0.03 |
|  | PMA | FGF | 121.96 | 91.09 | 0.04 |
|  | PMA | IL13 | 1639.03 | 420.71 | 0.01 |
|  | PMA | IL3 | 890.70 | 193.40 | 0.01 |
|  | PMA | IL4 | 873.80 | 280.20 | 0.003 |
|  | PMA | IL5 | 404.40 | 62.70 | 0.0006* |

***** Remains significant after adjustment for multiple comparisons (Bonferroni adjusted *p-*value = 0.0012)

EOI: end of induction; PMA: phorbol myristate acetate/ionomycin, SP: *S. pneumonia*

**Supplementary Table 1: Summary of significant cytokine values for patients with and without infection, by stimulant-cytokine combinations and sample period.**

| **Sample period** | **Stimulant** | **Cytokine** | **Cytokine values (pg/ml)** | | ***P* value** |
| --- | --- | --- | --- | --- | --- |
|  |  |  | **Infection** | **No infection** |  |
| Baseline | SP | IL13 | 0.45 | 0.01 | 0.05 |
| EOI | CMV | RANTES | 528.71 | 174.55 | 0.01 |
|  | Control | IFNγ | 2.15 | 0.67 | 0.02 |
| Maintenance | CMV | MCP1 | 8958.63 | 7622.01 | 0.03 |
|  | Control | MCP1 | 9220.46 | 7176.75 | 0.013 |
|  | PMA | IL13 | 1923.87 | 487.65 | 0.006 |
|  | PMA | IL3 | 1728.05 | 202.50 | 0.013 |
|  | PMA | IL4 | 870.17 | 312.91 | 0.0095 |
|  | PMA | IL5 | 405.43 | 66.69 | 0.0003* |

***** Remains significant after adjustment for multiple comparisons (Bonferroni adjusted *p-*value = 0.0012)

EOI: end of induction; SP: *S. pneumonia,* CMV: cytomegalovirus, PMA: phorbol myristate acetate/ionomycin

**Supplementary Table 2: Summary of significant cytokine values for patients with and without clinically-defined and microbiologically-defined infections, by stimulant-cytokine combinations and sample period.**

| **Sample period** | **Stimulant** | **Cytokine** | **Cytokine response ratio*** | | ***P* value** |
| --- | --- | --- | --- | --- | --- |
|  |  |  | **Infection** | **Non-infection** |  |
| Maintenance | PMA:unstimulated | IL 2 | 144498 | 1075215 | 0.03 |
|  | PMA:unstimulated | IL 3 | 87927 | 9210 | 0.01 |
|  | PMA:unstimulated | IL 5 | 25063 | 3793 | 0.0015 |
|  | PMA:unstimulated | IL 9 | 13502 | 530 | 0.02 |
|  | PMA:unstimulated | IL 13 | 163903 | 10509 | 0.003 |
|  | PMA:unstimulated | GM-CSF | 1564094 | 121294 | 0.03 |

PMA: phorbol myristate acetate/ionomycin

*Ratio of cytokine values (pg/ml) of PMA-stimulated samples to cytokine values (pg/ml) from unstimulated (control) samples.

**Supplementary Table 3: Summary of significant cytokine response ratio (mitogen-stimulated: unstimulated samples) in patients with and without infection, by sample period**
